# Next-Generation Sequencing in Practical Wildlife Disease Investigation: Metagenomic Insights into European Bison and Gazelle Mortality and Morbidity Events

**DOI:** 10.3390/v18101118

**Published:** 2026-10-09

**Authors:** Wojciech Socha, Michał K. Krzysiak, Elżbieta Moniuszko, Paweł Kulik, Jarosław Tomana, Magdalena Larska

**Affiliations:** 1 Department of Virology and Viral Animal Diseases, National Veterinary Research Institute, Partyzantów 57, 24-100 Puławy, Poland; wojciech.socha@piwet.pulawy.pl; 2 Department of Evolutionary and Physiological Ecology, Faculty of Biology, University of Białystok, K. Ciołkowskiego 1J, 15-245 Białystok, Poland; m.krzysiak@uwb.edu.pl; 3 “Medicor” Veterinary Service, Zwycięstwa 26B, 15-703 Białystok, Poland; elaem111@gmail.com; 4 Zamość Zoological Garden, Szczebrzeska 12, 22-400 Zamość, Poland; procurase@wp.pl; 5 Veterinary Clinic, Jana Kilinskiego 3A, 43-200 Pszczyna, Poland; jarek@tomana.com

**Keywords:** Gazelle Dorcas, European bison, metagenomics, necrotic balanoposthitis, gammaherpesvirus, *Pasteurella multocida*, *Porphyromonas levii*

## Abstract

Wildlife disease investigations require diagnostic methods capable of detecting diverse and previously unrecognized pathogens. Metagenomic next-generation sequencing (mNGS) was applied to samples from three Dorcas gazelles (*Gazella dorcas osiris*) affected by sudden mortality and four European bison (*Bison bonasus*) with respiratory disease or necrotic balanoposthitis (NBP). No dominant bacterial species was detected in gazelles. In European bison, respiratory disease was associated with *Pasteurella multocida*, whereas NBP samples were dominated by *Porphyromonas levii* and other anaerobes. Dorcas gazelle samples contained mainly Retroviridae-related sequences of undetermined pathogenic significance. Parapoxvirus-related annotations were not confirmed by PCR, suggesting non-specific or host-related sequence similarity. Macavirus-related sequences were consistent with previous PCR findings and enabled reconstruction of an approximately 51.1 kb fragment of a divergent gammaherpesvirus genome. These findings demonstrate the value of mNGS for wildlife disease investigation and genomic surveillance, while emphasizing the need for independent validation of metagenomic taxonomic assignments.

## 1. Introduction

Wildlife disease investigations increasingly require rapid, comprehensive, and unbiased diagnostic approaches capable of identifying both known and unexpected pathogens. Traditional diagnostic workflows in wildlife medicine often rely on targeted assays, culture-based techniques, histopathology, or serology, which may fail when dealing with mixed infections, novel agents, degraded field material, or cases with unclear etiologies [1]. In free-ranging and captive wild animals, where mortality events are frequently multifactorial and sample quality is often suboptimal, these limitations can substantially hinder timely disease recognition and epidemiological interpretation. Recent advances in next-generation sequencing (NGS), particularly metagenomic sequencing, have transformed infectious disease diagnostics by enabling simultaneous detection of viral, bacterial, fungal, and parasitic nucleic acids without prior assumptions regarding pathogen identity [2,3,4]. Metagenomic approaches have become increasingly important in veterinary and wildlife surveillance because they permit characterization of complex microbial communities, detection of emerging pathogens, and identification of co-infections that may otherwise remain undiagnosed [5]. Viral metagenomics has been especially valuable for studying wildlife-associated pathogens and zoonotic reservoirs, contributing to the expanding understanding of global virome diversity and pathogen evolution [6]. This was found to be especially useful for detection of rare, previously unknown pathogens or in cases where clinical signs are not specific [7,8]. This is often the case for diseases in wildlife, as for many species of animals, knowledge of their pathogens remains limited. While many of those pathogens are species-specific, wildlife could also be a reservoir of infectious agents that could cause outbreaks in farmed animals or even humans [9]. Wild ungulates constitute an important epidemiological interface between wildlife, livestock, and human health. The European bison (*Bison bonasus*), Europe’s largest terrestrial mammal, represents a conservation-dependent species characterized by low genetic diversity and susceptibility to infectious diseases [10]. Despite successful restitution programs, European bison populations remain vulnerable to bacterial, viral, and parasitic infections that may affect both individual survival and long-term population stability [11]. Similarly, captive exotic ungulates such as gazelles are exposed to infectious threats associated with animal translocation, intensive management, environmental stressors, and interspecies pathogen transmission [12]. Disease outbreaks in these species therefore have important implications for conservation biology, zoo medicine, and transboundary disease surveillance. Metagenomic NGS offers unique opportunities in wildlife pathology because it enables retrospective and hypothesis-free investigation of morbidity and mortality events. Unlike targeted molecular methods, metagenomics can reveal unexpected etiological agents, characterize microbial dysbiosis, and identify mixed infections contributing to clinical deterioration [3,4,5]. Previous studies have demonstrated the utility of metagenomic sequencing in detecting respiratory, enteric, and systemic pathogens in domestic and wild animals, highlighting its value as a surveillance and diagnostic tool at the wildlife–livestock interface [2,6]. However, despite the growing use of metagenomics in veterinary medicine, reports describing its practical application in routine wildlife disease investigations remain limited. In particular, there is a need for case-based studies demonstrating how NGS-derived metagenomic data can support diagnostic interpretation in real-world wildlife morbidity and mortality investigations involving multiple species and diverse pathological presentations.

## 2. Materials and Methods

### 2.1. Sample Collection Overview

**Figure 1:**
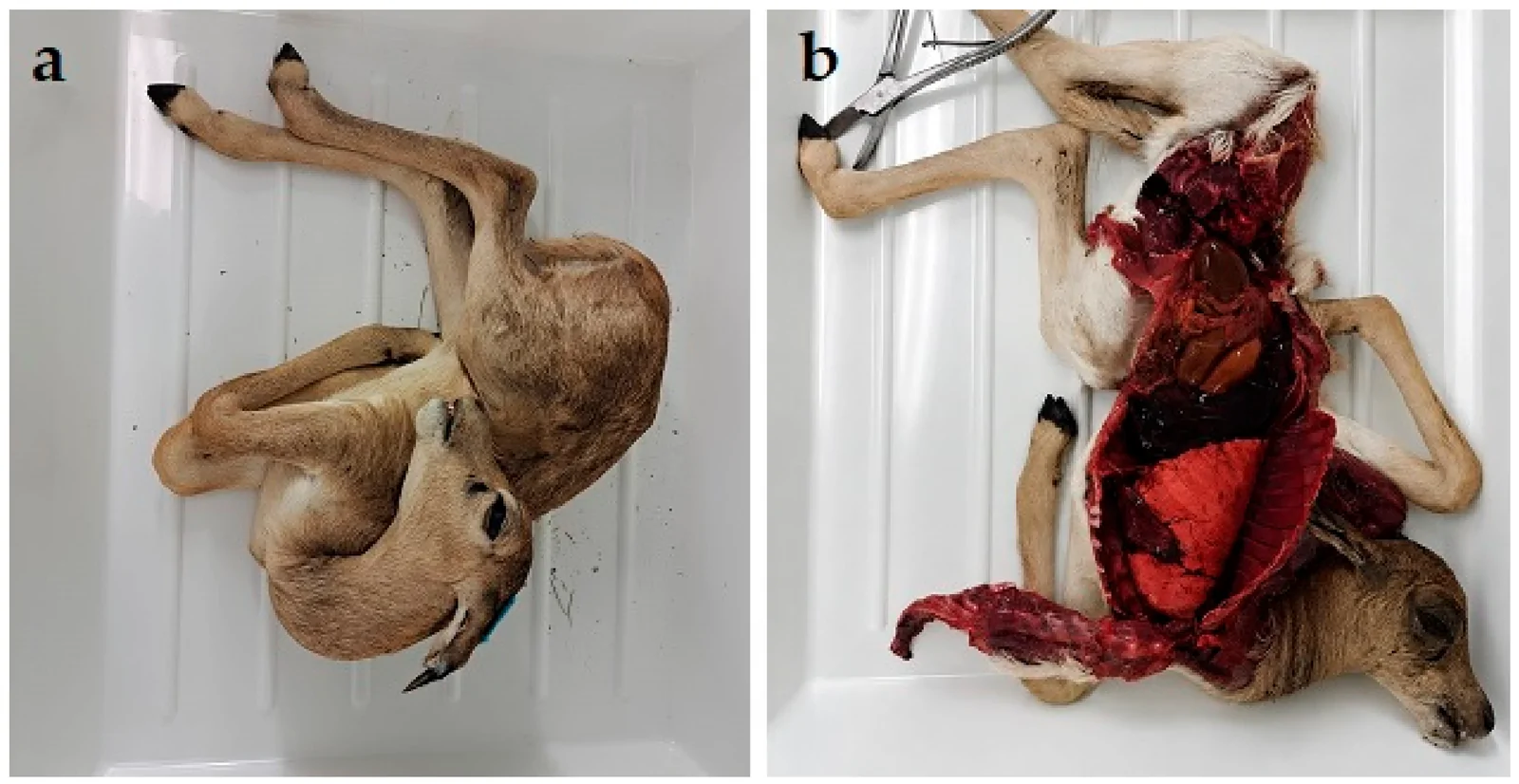
Juvenile dorcas gazelle (*Gazella dorcas osiris*) found dead during the outbreak investigation at the zoological garden in southeastern Poland. The animal was discovered in lateral recumbency without evident premortem clinical signs (**a**). Gross examination revealed severe diffuse congestion of the lungs, liver, and spleen, accompanied by multifocal serosal hemorrhages and generalized visceral hyperemia consistent with fulminant systemic inflammatory or vascular disorder (**b**).

The study used samples collected during three separate epizootic investigations. The first group of samples was collected from three dorcas gazelles (*Gazella dorcas osiris*) from an outbreak of sudden fatal disease at the Zamość Zoological Garden in southeastern Poland. During few months in 2023, deaths of six adult gazelles and three younger was recorded, leaving only a couple left alive. Most affected animals died peracutely and without evident premortem clinical manifestations or evident postmortem lesions. No intoxication, environmental or management-related stressors that could explain the mortality event were identified. The necropsies were performed on site immediately after the body was found or after it was frozen at −20 °C in the institute’s autopsy room (Figure 1). Each sample from dorcas gazelle was coded by number specifying the animal of origin and letter describing the specific tissue. In total, five samples were taken for the study, including three blood samples (A123K, A126K, A158K), a nasal swab (A126N), and a spleen (A123S) [13].

**Figure 2:**
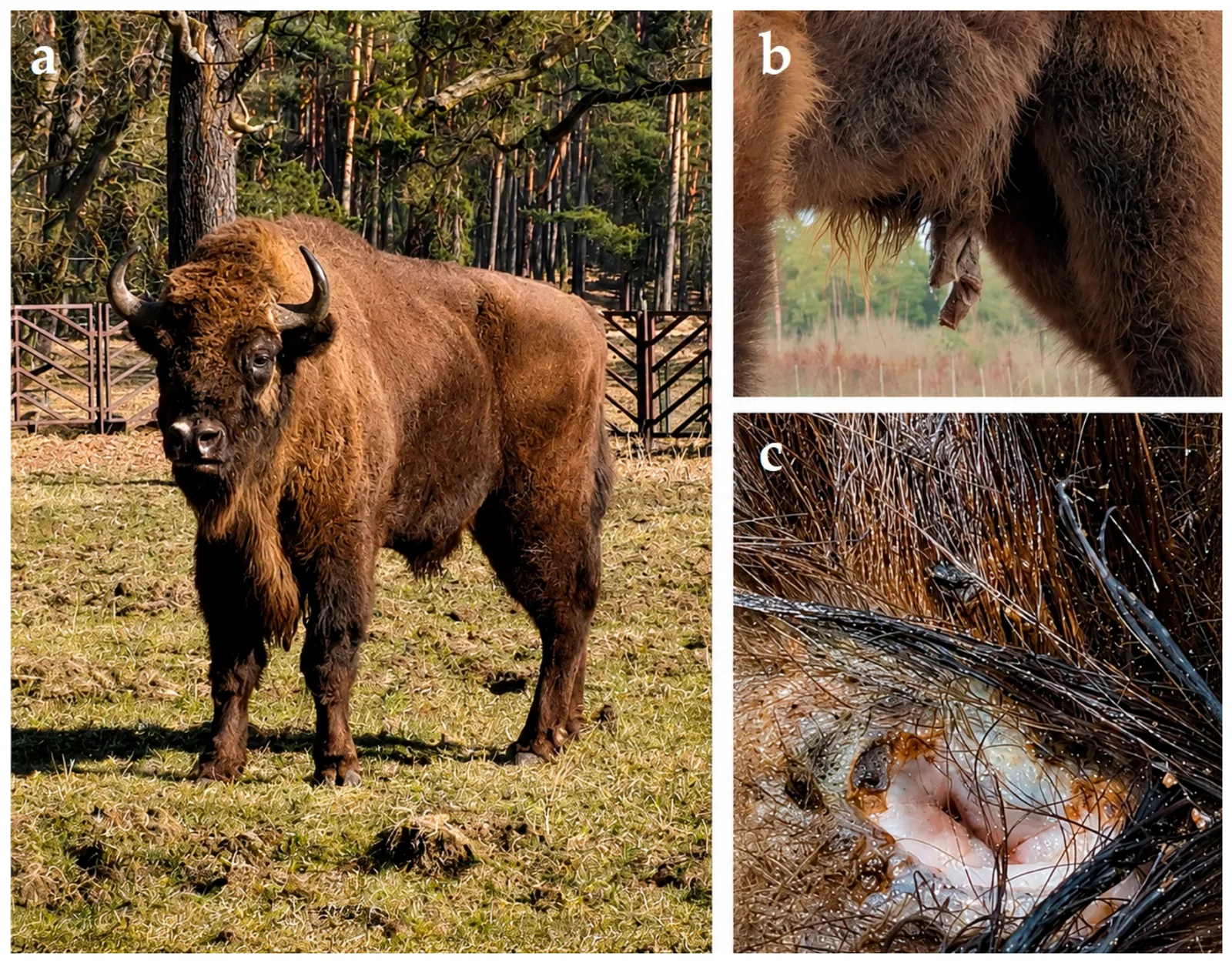
Various stages of necrotic balanoposthitis (NBP) in European bison (*Bison bonasus*) bulls in Pszczyna, Poland. (**a**) Marked edema of the lower abdomen and preputial region; (**b**) prolapse of necrotic penile tissues from the preputial opening and partial autoamputation; (**c**) ulcerative and necrotic lesions of the penile mucosa with purulent exudate and tissue destruction.

**Figure 3:**
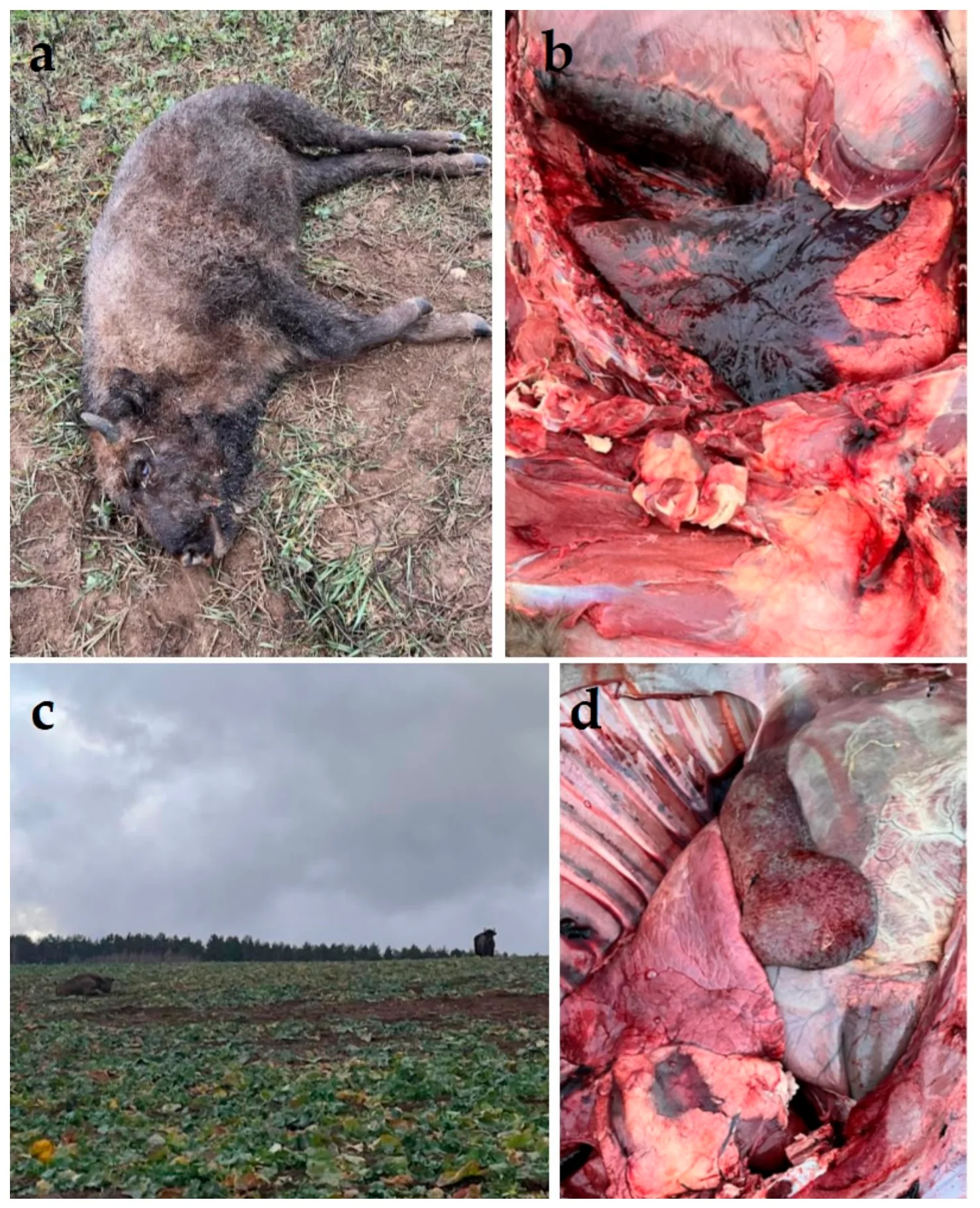
Gross findings in free-ranging European bison (*Bison bonasus*) found dead during field investigations. (**a**) carcass discovered in lateral recumbency without evidence of external traumatic injury (sample A1162); (**b**) with severe diffuse pulmonary congestion and edema; (**c**) site where the second carcass was identified in an agricultural field (sample A1163); (**d**) multifocal pulmonary congestion, generalized thoracic hyperemia, petechial hemorrhages of the serosal membranes and enlargement of the spleen. The lesions were consistent with acute systemic disease associated with severe circulatory disturbances in both animals.

The second investigation involved an outbreak of necrotic balanopostitis (NBP) in European bison (*Bison bonasus*) in the enclosure in Pszczyna, southern Poland, in 2022 [14]. Due to the severe progression of the disease, a decision was made to euthanize 7 bulls in 2023 and another 3 in 2024 (Figure 2). The culling was granted by the General Director for Environmental Protection on the basis of the Polish Nature Conservation Act of 16 April 2004, which allows derogations from strict species protection. The samples of foreskin fragments were collected immediately after euthanasia from two affected European bison bulls (A1018, A1019). The remaining third set of samples derived from the lungs of two young free-ranging European bison (A1162, A1163) from the Knyszyńska Forest population in the north-east of Poland (Figure 3). These animals were among nine other European bison found dead in the same area during the winter of 2024/2025. Autopsy performed on site confirmed that death occurred shortly (12–24 h) before sampling. All the samples were stored at −20 °C until testing.

### 2.2. Sample Preparation and DNA/RNA Extraction

Prior to DNA/RNA extraction, the samples were prepared as follows: the whole blood was diluted in PBS without calcium and magnesium (MP Biomedicals) in a proportion of 1:3; “dry” nasal swabs were soaked in 1 mL Minimum Essential Medium Eagle (MEM) (Sigma-Aldrich, Irvine, UK) for a minimum of 6 h at 18–25° and mixed. In case of tissue samples, 10% homogenates were prepared in MEM with 1.4 mm ceramic beads (Lysing Matrix D, MP Biomedicals, Irvine, CA, USA) using a TissueLyser LT (Qiagen, Hilden, Germany). Genetic material was extracted from 200 µL of samples using a IndiMag Pathogen Kit w/o plastics (INDICAL Bioscience, Leipzig, Germany) according to the manufacturer’s guidelines.

### 2.3. Library Preparation and Sequencing

Genetic material extracted from samples was sent to Novogene Gmbh (Munich, Germany) for library preparation and high-throughput sequencing. Before sequencing, all samples were tested for quantity and quality of DNA. Only samples that contained a minimum of 100 ng of total DNA with a concentration over 5 ng/μL and high purity (OD260/280 = 1.8–2.0) were subject to sequencing. Additionally, samples where most of the DNA was fragmented below 500 bp were eliminated from further processing (Table S1). Samples that passed quality control were randomly fragmented into approximately 350 bp long fragments using a Covaris ultrasonic disruptor to construct the library. Resulting fragments were end-repaired, A-tailed, and ligated with sequencing adapters. Fragments with adapters were size-selected and PCR-amplified. The library was quantified using Qubit and qPCR, and the size distribution was detected with a fragment analyzer. After passing the quality check, different libraries were pooled according to their effective concentrations and target data output requirements and then subjected to PE150 sequencing on the Illumina NovaSeq X Plus platform (Illumina, San Diego, CA, USA).

### 2.4. Bioinformatic Analysis

#### 2.4.1. Data Processing

The initial sequencing data processing and metagenomic annotation were performed by Novogene GmbH as part of the contracted sequencing and bioinformatics service. Briefly, raw sequencing data were preprocessed with Fastp 0.23.1 using parameters: -g -q 5 -u 50 -n 15 -l 150 --overlap_diff_limit 1 --overlap_diff_percent_limit 10 to eliminate reads containing adapters and low-quality reads. For *Bison bonasus* samples, host-derived reads were filtered against the European bison reference genome (GCA_963879515.1) using Bowtie2 2.5.4 with the following parameters: --end-to-end --sensitive --no-hd --no-sq -I 200 -X 400 --threads 8. For *Gazella dorcas*, host read removal against a *G. dorcas* reference genome was not performed because a suitable reference was not available. Sequencing and quality-control statistics, including raw and clean sequencing data and the amount of data remaining after host-read removal, are provided in Supplementary Table S1. Metagenomic assembly was performed using MEGAHIT 1.2.9 with the meta-large preset and the parameters --presets meta-large -m 180000000000 -t 2. Following assembly, scaftigs shorter than 500 bp were discarded, and scaftigs of ≥500 bp were retained for subsequent gene prediction and annotation. Gene prediction was performed using GeneMark.hmm 2.1 with the parameters -a -d -f G -p 1, and predicted ORFs shorter than 100 amino acids were excluded. The remaining ORFs were dereplicated using CD-HIT v4.5.8 at 95% sequence identity and 90% coverage to generate a non-redundant gene catalog. Clean reads were subsequently mapped to the gene catalog using Bowtie2 2.5.4 with the same parameters described above to estimate gene abundance. DIAMOND v2.1.9 [14] was used to align the predicted gene sequences against the MicroNR database (version 2024.03), which contains microbial sequences from bacteria, fungi, archaea, and viruses extracted from the NCBI non-redundant (NR) protein sequence database. The alignment was performed using the parameters -p 4 -e 1e-5 -k 50 --id 30 --sensitive. Taxonomic assignments were subsequently determined using the lowest common ancestor (LCA) algorithm implemented in MEGAN [15]. Taxonomic abundance tables were generated at the kingdom, phylum, class, order, family, genus, and species levels. Species abundance was calculated as the sum of the abundances of genes assigned to the respective species [16,17,18]. These abundance tables were subsequently used for taxonomic profiling, including Krona visualization and relative-abundance analyses [19].

#### 2.4.2. Gene Abundance Analysis

Taxonomic annotation tables generated by Novogene GmbH at the family and genus level were screened to identify viral/bacterial taxa detected in each analyzed sample. Gene annotation counts were used to identify dominant viral/bacterial taxa, whereas gene abundance matrices (RPK-normalized values) served as an auxiliary criterion for prioritizing samples and viral candidates for downstream analyses, including comparative genome mapping, genome reconstruction and phylogenetic characterization.

#### 2.4.3. Comparative Genome Mapping and Viral Sequence Validation

Assembled scaffold sequences from each analyzed sample were initially screened for sequence similarity to viral references using local BLASTN-based similarity searches against selected Macavirus and Poxviridae reference genomes. Viral reference sequences were used to construct local nucleotide databases with makeblastdb, and scaffold-to-reference searches were performed using BLAST+ v2.17.0. The final BLASTN searches were performed using the blastn task with an E-value threshold of 1, a word size of 7, a match reward of 1, a mismatch penalty of −1, a gap opening penalty of 2, and a gap extension penalty of 1. Eight computational threads were used. BLAST results were subsequently filtered using an R-based procedure in R version 4.3.0 and RStudio 2026.01.1 (Build 403), retaining alignments of at least 300 bp in length and a minimum sequence identity of 65%. The resulting alignments were used to identify viral sequence candidates and assess the distribution of homologous regions within the assembled scaffolds and among analyzed samples.

#### 2.4.4. Macavirus Genome Reconstruction, Sequence Validation and Phylogenetic Analysis

Samples showing the strongest Macavirus-related signal based on taxonomic annotation and gene abundance data were selected for detailed downstream analysis. To identify homologous regions and assess the suitability of available reference genomes for comparative mapping, the assembled scaffolds from sample A126N were aligned against three Macavirus reference genomes: PV553530.1 (Macavirus sp. isolate pool43), PX942233.1 (Macavirus ovinegamma2 isolate DGYB/97548) and PV231823.1 (OvHV-2 isolate TW01), using the BLASTN-based comparative mapping approach described above. The resulting scaffold-to-reference alignments were evaluated using the filtering criteria described earlier to assess the extent and distribution of homologous regions across the three reference genomes. PX942233.1 provided the most consistent and extensive homologous regions at the genomic loci selected for downstream analysis, including the glycoprotein B, DNA polymerase and helicase coding regions, and was therefore selected as the primary reference for subsequent viral genome reconstruction. Genomic regions supported by BLASTN alignments meeting the predefined length and identity thresholds were extracted from the assembled scaffolds and compared with homologous reference sequences. Overlapping alignments were consolidated to define continuous reconstructed regions when supported by the scaffold sequences. Regions lacking sufficient sequence evidence were not inferred directly from the reference sequence and were retained as unresolved regions without specific gene annotation. Scaffold alignments were manually inspected in relation to annotated genomic features to assess the distribution of homologous regions across the reference sequence. Following genome reconstruction, raw paired-end reads from sample A126N were independently mapped to the reconstructed viral sequence using Bowtie2 in Unipro UGENE v52.1 to assess read-level support for the reconstructed genomic regions. Per-base coverage was exported from UGENE and subsequently analyzed in R version 4.3.0 using RStudio 2026.01.1 (Build 403). Mean read depth was calculated for each annotated CDS based on the corresponding coordinates of the reconstructed sequence.

Selected fragments of the Macavirus genome, encoding glycoprotein B, DNA polymerase and helicase, were aligned using ClustalW implemented in MEGA 12.1.1 software. Alignments were manually trimmed to retain comparable genomic regions and to allow the inclusion of an appropriate set of reference sequences. Neighbor-joining phylogenetic trees were subsequently constructed using the p-distance method, with transitions and transversions included and pairwise deletion of gaps and missing data. The reliability of the inferred phylogenetic relationships was assessed using the bootstrap method with 500 replicates.

### 2.5. Confirmatory PCRs

To confirm results of metagenomic NGS, PCRs specific to herpesviruses and poxviruses were performed. Panherpesvirus-specific nested PCR was already described previously [13] using primers flanking fragments of the viral DNA polymerase (DPOL) gene designed by Van Devanter et al., 1996 [15]. The Orf virus (ORFV) specific PCR was used to confirm the presence of the virus in samples from dorcas gazelle and European bison. Two sets of primers were used: one specific to viral interleukin 10 (vIL-10) orthologue, and the second specific to interleukin-2 inhibition factor (GIF), using modified procedures described by Klein et al., 2005 (Table 1) [16]. The reaction was run using JumpStart™ REDTaq^®^ DNA Polymerase (Sigma, St. Louis, MO, USA). The reaction mix consisted of 17.5 μL of water, 2.5 μL of reaction buffer, 1 µL of each primer (vIL-10-3:vIL-10-4 for vIL-10 specific reaction; GIF5:GIF6 for GIF) (10 μM), 0.5 μL of dNTPs (10 μM) and 2 μL of DNA sample. Reaction conditions included 15 min preincubation at 95 °C followed by two rounds of amplification. The first 5 cycles of amplification consisted of 30 s denaturation at 94 °C, 2 min annealing at 57 °C and 30 s elongation at 72 °C. The next 35 cycles of amplification consisted of 30 s denaturation at 94 °C, 30 s annealing at 57 °C and 30 s elongation at 72 °C. The reaction was ended with 10 min incubation at 72 °C. A total of 5 μL of each PCR product was visualized on 1.5% agarose in 1× Tris-acetate-EDTA (TAE). Electrophoretic separation was carried out for 30 min at 90 V. The molecular weight of the obtained products was assessed using a GeneRuler Low Range DNA Ladder (Thermo Fisher Scientific, Waltham, MA, USA). Positive samples were visible at 408 bp for the GIF5:GIF6 primer pair and 300 bp for vIL-10-3:vIL-10-4.

### 2.6. Generative AI Assistance

Generative artificial intelligence tools (ChatGPT, OpenAI, San Francisco, CA, USA; https://openai.com/chatgpt (accesed on 1 September 2026) based on the GPT-5 family of large language models were used to assist with bioinformatic workflow optimization, interpretation of sequence analysis outputs, troubleshooting of computational procedures, and language refinement during manuscript preparation. Final analytical decisions, biological interpretations of the results, and data verification were independently performed by authors.

## 3. Results

All collected samples passed DNA quality control tests and were successfully processed for metagenomic sequencing (Table S2). Following initial preprocessing and removal of host-derived reads, kingdom-level taxonomic annotation tables were generated for each sample. Dorcas gazelle samples were dominated by unassigned annotations (49.25–52.08%), followed by bacterial (43.37–48.04%) and viral annotations (2.36–2.65%), with the total number of gene annotations ranging from 176,166 to 197,318. European bison samples contained fewer gene annotations overall (10,496–15,834) and were similarly dominated by bacterial (86.84–89.35%) and unassigned (10.21–12.48%) annotations, whereas viral annotations represented only a minor fraction (0.22–0.56%) (Table S3).

### 3.1. Virological Investigation

**Figure 4:**
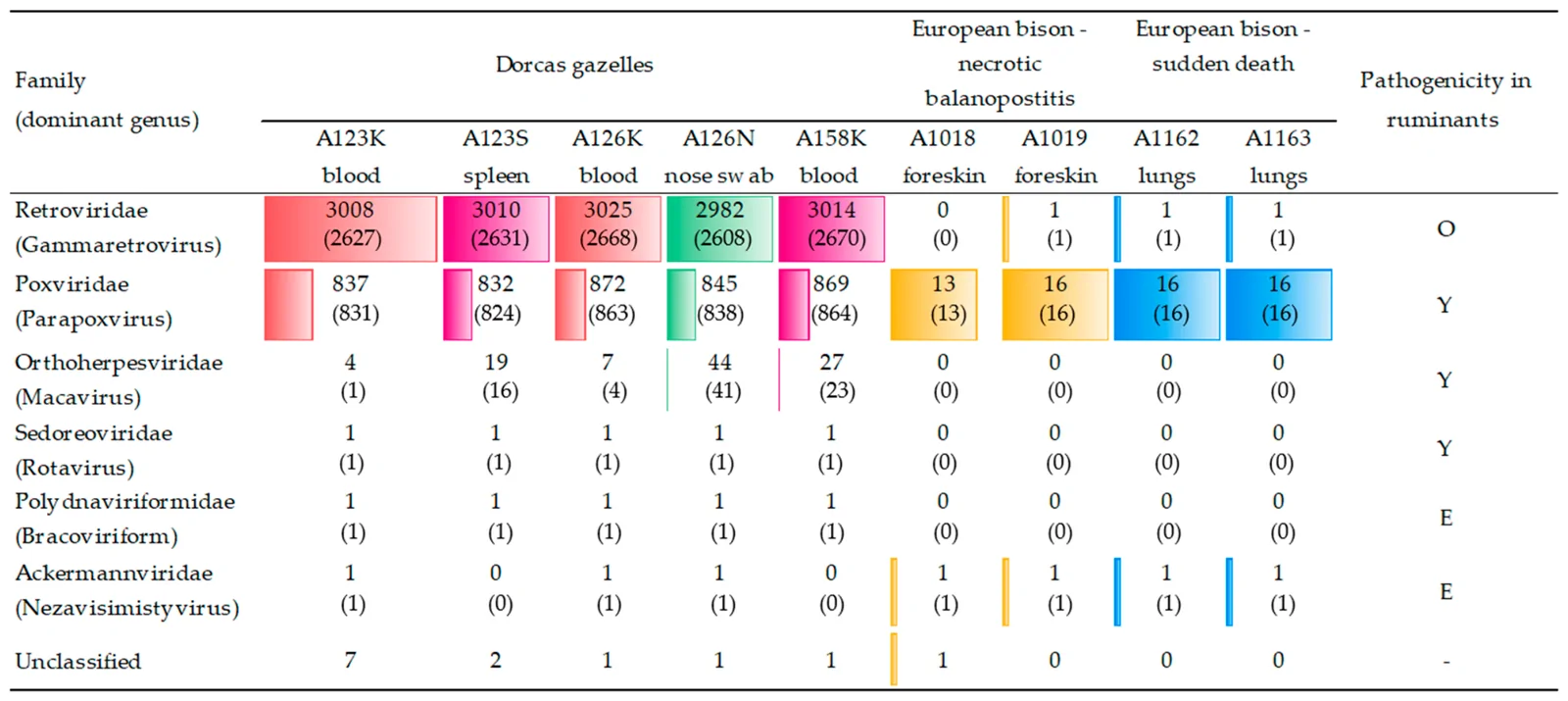
Distribution of dominant viral families and genera identified by metagenomic sequencing in dorcas gazelles (A123K, A123S, A126K, A126N, and A158K originated from gazelles that died during a mortality event) and European bison (samples A1018 and A1019 were collected from animals affected by necrotic balanoposthitis (NBP), whereas samples A1162 and A1163 originated from animals found dead with signs of pneumonia). Horizontal bars represent the number of taxon-specific viral gene annotations. Annotations from each sample are coded with different color. Viral taxa were classified according to their reported pathogenic potential in ruminants as pathogenic (Y), opportunistic (O), or environmental (E) based on the available literature [17,18,19,20,21]. “Unclassified” represents viral-gene annotations that could not be confidently assigned to a defined viral genus or family.

The relative representation of viral taxa was assessed using genus-level taxonomic annotation counts. Family-level annotation counts were calculated as the sum of annotations assigned to all genera belonging to the corresponding viral family. An additional category, designated as “unclassified” was added to include viral gene annotations that could not be confidently assigned to a defined viral family or genus. Overall, the number of viral gene annotations was substantially higher in samples collected from Dorcas gazelle (3859–3913) than in European bison samples (15–18). Viral gene annotations detected in Dorcas gazelles were dominated by members of the families Retroviridae, Poxviridae and Orthoherpesviridae, whereas Parapoxvirus was the only viral genus consistently predominant in all European bison samples (Figure 4).

Gammaretrovirus gene annotations constituted the most abundant viral category in all analyzed Dorcas gazelle samples (1843–1894 annotated genes per sample). Taxonomic assignments were distributed across numerous gammaretrovirus taxa represented by sequences associated with different mammalian hosts, including rodents, bats, carnivores, primates and cervids. The annotations included taxa associated with both exogenous gammaretroviruses and endogenous retrovirus-related sequences (Table S4). These annotations were considered more likely to represent endogenous retroviral elements than exogenous infection and could not be directly linked to the observed clinical signs; therefore, no further analyses were performed. Gene annotations assigned to *Macavirus* and *Parapoxvirus* were less abundant but were selected for further downstream analyses because of their recognized pathogenic potential in ruminants. Poxviridae gene annotations were identified in both Dorcas gazelle and European bison samples. In European bison, all identified Poxviridae gene annotations were assigned exclusively to ORFV, whereas in Dorcas gazelles, additional annotations were assigned to representatives of *Avipoxvirus*, *Orthopoxvirus* and *Yatapoxvirus*.

In the subsequent sequence-level analysis, most Poxviridae-related BLASTN matches corresponded to regions annotated as encoding three proteins (PP1, PP287 and PP288) derived from ORFV strain D1701 (HM133903.1) in two specific regions of the viral genome, with no evidence of additional homologous regions. One of these regions displayed strong similarity to mammalian genomic sequences, raising the possibility of host-derived cross-homology. To independently assess the presence of ORFV DNA, PCR assays targeting the vIL10 and GIF genes were performed. No amplification products were obtained from any analyzed sample. Together, the comparative mapping and PCR results did not provide sufficient evidence to support the presence of an authentic Poxvirus infection in either Dorcas gazelles or European bison.

**Figure 5:**
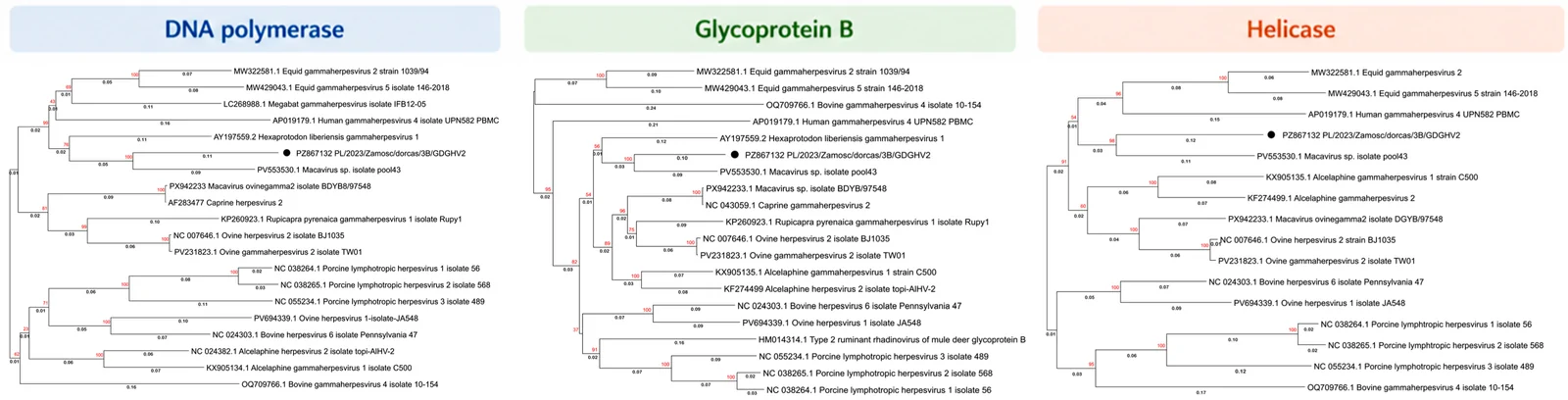
Neighbor-joining phylogenetic tree based on partial sequences of three genomic regions of A126N (● PZ867132 PL/2023/Zamosc/Dorcas/3B/GDGHV2): DNA polymerase (666 bp), glycoprotein B (692 bp) and helicase (396 bp). Bootstrap support values based on 500 replicates are shown in red. Branch lengths are shown for values ≥ 0.01.

Gene annotations assigned to the genus Macavirus were detected in several Dorcas gazelle samples. This was consistent with previous PCR and Sanger sequencing results, which detected herpesvirus-specific sequences in the analyzed gazelle samples [12]. Among positive samples, A126N had the highest number of gene annotations assigned to Macavirus and was therefore selected for detailed downstream analyses. Among the evaluated reference sequences, PX942233.1 (Macavirus ovinegamma2 isolate DGYB/97548) provided the most consistent and extensive homologous regions in the genomic *loci* selected for downstream analysis and was therefore used as the primary reference for reconstruction. Comparative mapping identified multiple homologous regions distributed across the reference sequence and supported the partial reconstruction of a divergent *Macavirus*–like genome. As a result, a partial genomic reconstruction of Gazella dorcas gammaherpesvirus 2 isolate PL/2023/Zamosc/dorcas/3B/GDGHV2 was obtained and deposited in NCBI GenBank under accession number PZ867132. The reconstruction was represented on the 97,548 bp coordinate system of PX942233.1, with 51,140 bp (52.4%) corresponding to defined nucleotide positions. The reconstruction was derived from 7 metagenomic scaffolds covering discontinuous regions of the reference sequence and contained 16 annotated viral CDS, including 13 CDS reconstructed across their complete extent and 3 partial CDS with unsupported terminal regions (Table S5). The reconstructed sequence PZ867132 was subsequently used as the reference for an independent read-level assessment. Mapping of the raw A126N reads showed that all 16 annotated CDS were covered across their entire defined nucleotide ranges, with mean read depths ranging from 469x to 551x (Table S5). Phylogenetic analysis of the DNA polymerase, glycoprotein B and helicase regions consistently placed the detected virus within a distinct *Macavirus*-related lineage, closely related to *Macavirus* sp. isolate pool43 (PV553530.1), a gammaherpesvirus identified in blue duiker from the Democratic Republic of the Congo [22]. This relationship was strongly supported by high bootstrap values for each analyzed gene: DNA polymerase (100%), glycoprotein B (100%) and helicase (98%) (Figure 5).

### 3.2. Bacterial Metagenomics

**Figure 6:**
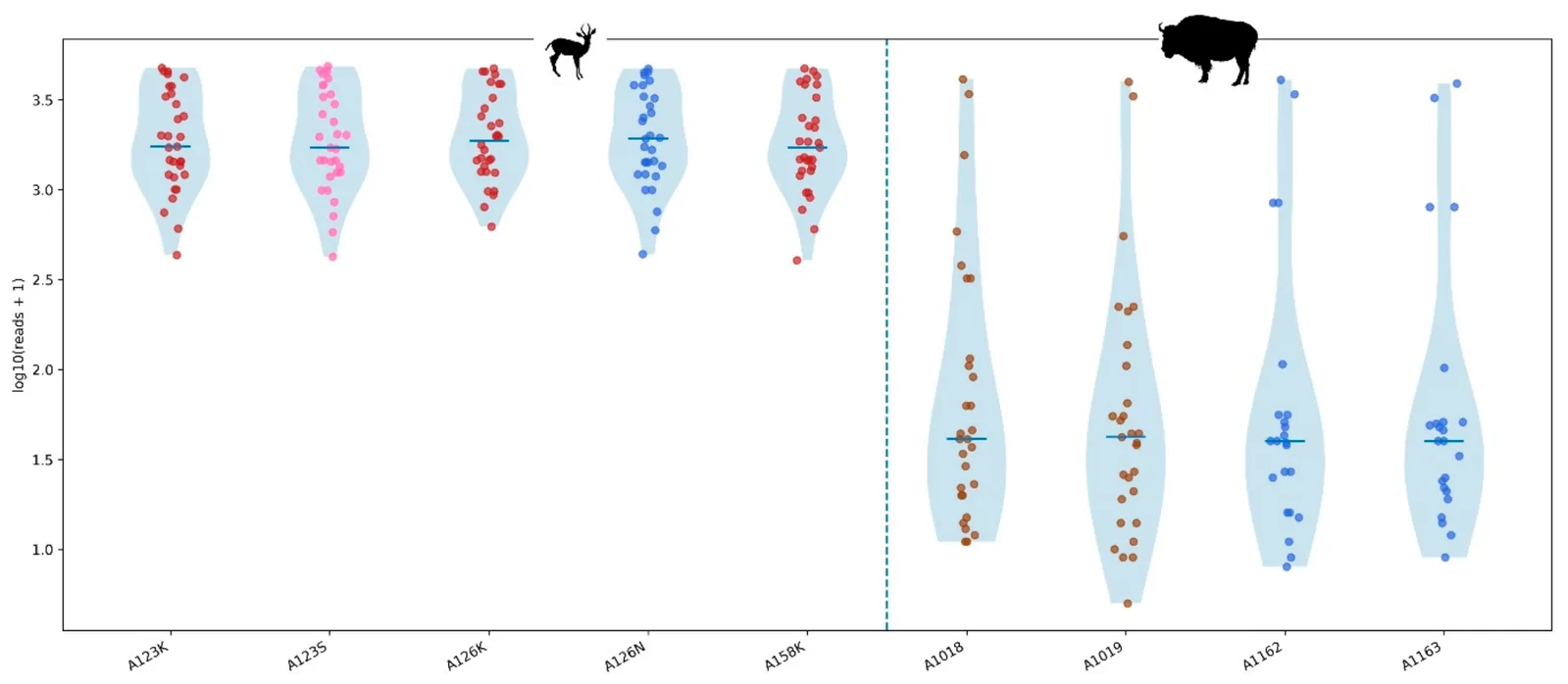
Violin plots showing the distribution of log-transformed bacterial gene annotation abundances obtained by metagenomic sequencing of samples collected from dorcas gazelles and European bison. Individual points represent bacterial genera detected in each animal sample, while violin shapes illustrate the density distribution of bacterial abundance profiles. Gazelle samples A123K, A126K, and A158K are shown in red, A123S in pink, and A126N in blue. European bison samples A1018 and A1019 are indicated by brown points, whereas A1162 and A1163 are shown in blue.

The relative representation of bacterial taxa was assessed based on species-level annotation tables. Metagenomic analysis revealed marked differences in the distribution of bacterial annotation abundances between the analyzed samples (Figure 6). Gazelle samples showed relatively similar distributions of bacterial gene annotations, whereas European bison samples exhibited greater inter-sample heterogeneity.

**Figure 7:**
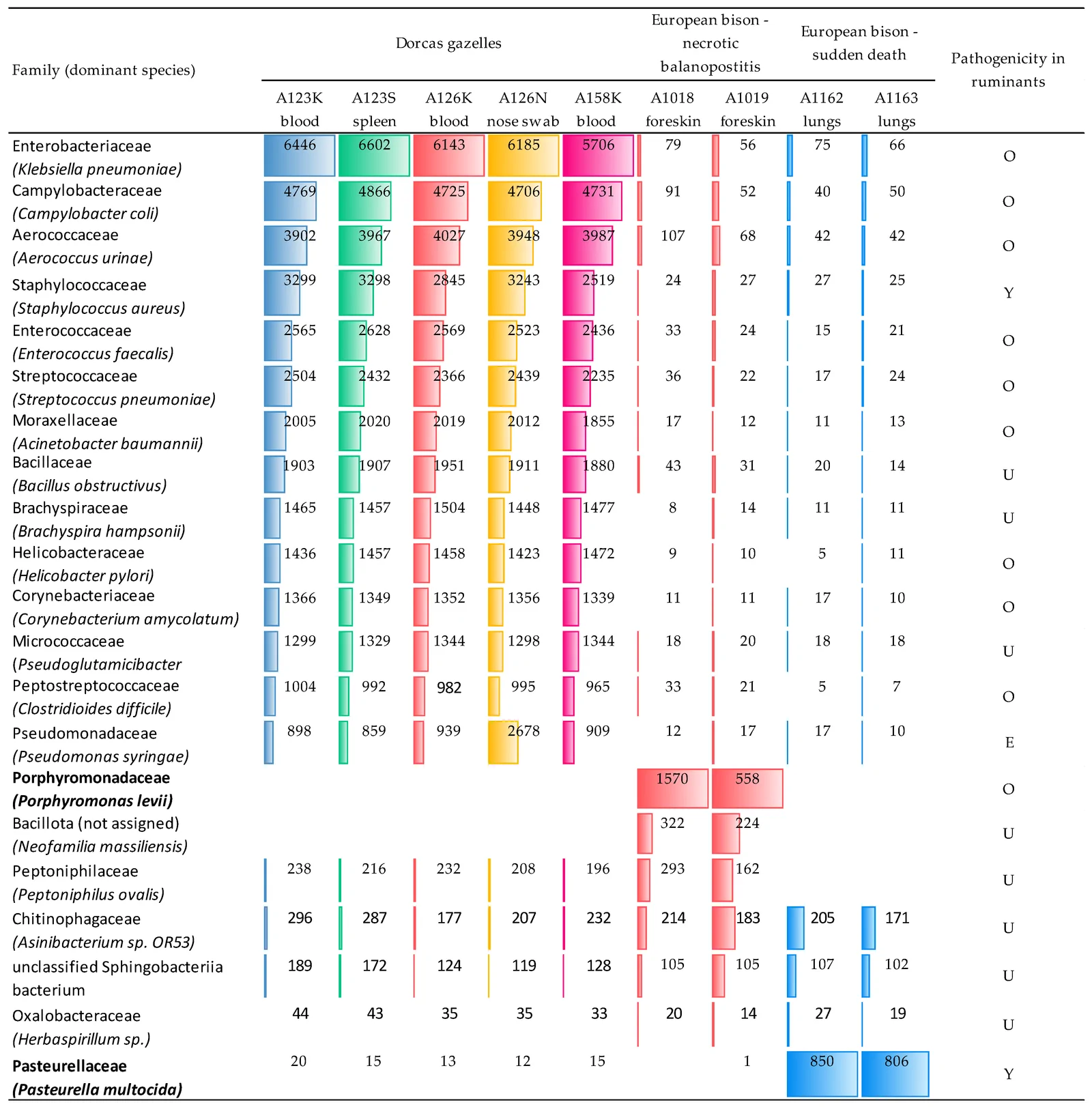
Distribution of dominant bacterial families and representative species identified by metagenomic sequencing in dorcas gazelles (A123K, A123S, A126K, A126N, and A158K originated from gazelles that died during a mortality event) and European bison (samples A1018 and A1019 were collected from animals affected by necrotic balanoposthitis, whereas samples A1162 and A1163 originated from animals found dead with signs of pneumonia). Horizontal bars represent the number of taxon-specific metagenomic annotations. Annotations from each sample are coded with different color. Bacterial taxa were classified according to their reported pathogenic potential in ruminants as pathogenic (Y), opportunistic (O), environmental (E), or of unknown/insufficiently documented pathogenic potential (U), based on the available literature [22,25,26,27,28,29,30,31,32,33]. In two cases, more than one dominant species was identified, including *Corynebacterium diphtheria* and *Peptoniphilus* sp. ING2D1G. In bold are marked predominant species of *Porphyromonas levii* and *Pasteurella multocida*, the presence of which is highly suggestive in relation to the clinical picture observed in the two European bison groups.

Bacterial annotations in Gazelle samples were predominantly assigned to members of the families Campylobacteriaceae, Enterobacteriaceae, Aerococcaceae, Staphylococcaceae, Enterococcaceae, and Streptococcaceae (Figure 7). The taxa with the highest numbers of bacterial gene annotations included *Campylobacter coli*, *Klebsiella pneumoniae*, *Aerococcus urinae*, *Staphylococcus aureus*, *Enterococcus faecalis*, and *Streptococcus pneumoniae*. In addition, a high number of gene annotations were also observed for several other bacterial taxa, including *Acinetobacter baumannii* (Acinetobacteraceae), *Brachyspira hampsonii* (Brachyspiraceae), *Helicobacter pylori* (Helicobacteraceae), *Clostridioides difficile* (Peptostreptococcaceae), *Pseudomonas syringae* (Pseudomonadaceae), and *Corynebacterium* spp. The relatively homogeneous bacterial profiles observed among gazelles indicate similarities in the bacterial taxa detected across these samples. These similarities may be consistent with exposure to a common infectious or dysbiotic process, but this interpretation remains uncertain given the potential effects of delayed necropsy and postmortem decomposition.

In European bison, substantially fewer bacterial gene annotations and a more heterogeneous bacterial taxonomic profile were observed. Samples collected from bulls affected with NBP (A1018 and A1019) were characterized by predominance of Porphyromonadaceae represented mainly by *Porphyromonas levii*, accompanied by Peptoniphilaceae (*Peptoniphilus ovalis* and *Peptoniphilus* sp. ING2D1G) and *Neofamilia massiliensis*. The observed bacterial profile is consistent with the involvement of bacterial taxa in the microbial communities associated with necrotic genital lesions in cattle and European bison [22,23,24]. In contrast, samples from European bison found dead (A1162 and A1163) demonstrated marked predominance of Pasteurellaceae represented by *Pasteurella multocida*. This finding supports *Pasteurella multocida* as a candidate bacterial contributor to the observed respiratory disease, although the metagenomic data alone do not establish a causal relationship. Several environment-associated taxa without clear evidence of primary pathogenic significance, including unclassified Sphingobacteriia bacterium, *Asinibacterium* sp. OR53, *Sediminibacterium* sp., and *Herbaspirillum huttiense*, were represented predominantly in European bison samples but by substantially fewer gene annotations (Figure 7).

## 4. Discussion

In this study, shotgun DNA metagenomic sequencing was applied to samples collected during three wildlife disease investigations representing two different diagnostic scenarios. In Dorcas gazelles, herpesvirus DNA had previously been detected by targeted PCR, allowing mNGS to be evaluated as a complementary approach for broader pathogen detection and genomic characterization [12]. In European bison, the causes of necrotic balanoposthitis and fatal respiratory disease were less clearly defined, and mNGS was used primarily as an exploratory diagnostic method. These contrasting applications illustrate both the strengths and limitations of metagenomics in practical wildlife disease investigation.

This approach was especially useful for samples from European bison exhibiting signs of NBP–diseases with complicated and uncertain etiology. NBP is one of the most threatening diseases affecting European bison bulls, with an annual prevalence of approximately 6% in the endemic area of Białowieża Forest, where it has been reported since the 1980s [24,34]. Despite decades of investigation, its etiology remains unresolved and is considered multifactorial. Several bacterial species, including *Trueperella pyogenes*, *Trueperella bialowiezensis*, *Fusobacterium necrophorum*, and *Mycoplasma bovigenitalium*, have been associated with lesions, although none has been consistently identified as the primary etiological agent [35,36,37]. Environmental contamination, repeated mucosal injury, host immune status, and the reduced genetic diversity characteristic of European bison populations have also been proposed as factors predisposing affected animals to disease development and progression [38,39]. The chronic and recurrent nature of the lesions, together with the inconsistent isolation of bacterial pathogens, suggests that additional infectious agents may contribute to disease pathogenesis. In that case, the use of metagenomic analysis as a way of detecting potential pathogens could be the most practical approach.

The results of the investigation of Dorcas gazelle mortality revealed a substantial representation of viral gene annotations. The highest number of viral gene annotations was assigned to members of the genus Gammaretrovirus (Table S3). These sequences may have originated from either exogenous viruses or endogenous retroviral elements integrated into the host genome [40,41]. Biological interpretation of these results is challenging for two reasons. First, the virome and genome–associated retroviral diversity of members of the genus Gazella is poorly characterized, which complicates the assignment of those sequences to known viral taxa. Second, for Gazella dorcas, host-derived reads were not removed during the initial bioinformatic processing because a suitable host reference genome was not available to the sequencing provider. As a result, endogenous viral elements (EVEs), which are integrated into the host genome, could have been retained among non-host reads and subsequently assigned to viral taxa [42]. On the other hand, although exogenous gammaretroviruses are suspected to be associated with immunosuppression, neoplasia, and increased susceptibility to secondary infections in mammals, the presence of viral sequences alone does not establish a causal role in disease [43]. Therefore, the pathogenic relevance of these sequences could not be assessed. In contrast, gene annotations assigned to retroviruses were almost completely absent from the European bison sample. Given that both endogenous and exogenous retroviruses have previously been described as widespread in bovids, this observation is unlikely to reflect a true absence of retroviral sequences [17,44]. This difference should be interpreted with caution because host-derived reads were processed differently between the two host species: reads from *Gazella dorcas* were not subjected to host-read removal, whereas reads from *Bison bonasus* were filtered against the European bison reference genome. The higher representation of retroviral annotations in gazelle samples may therefore partly reflect the retention of endogenous retroviral sequences originating from the host genome.

Detection of Macavirus genome fragments in the metagenomic analysis of Dorcas gazelle samples that had previously tested positive by panherpesvirus-specific PCR and Sanger sequencing [13], provided further sequence-based evidence for the presence of the virus. In the present study, metagenomic sequencing enabled a more extensive characterization of the virus, including analysis of a larger fragment of the DNA polymerase gene and two additional viral genes encoding glycoprotein B and helicase. Phylogenetic analysis of each of those fragments confirmed that all of them showed high homology with genes of gammaherpesvirus found in blue duiker in the Democratic Republic of the Congo [45]. The concordant topology obtained by metagenomic analysis from all three gene regions supports the robustness of this phylogenetic placement and suggests that the detected virus represents either a previously undescribed *Macavirus* or a divergent variant of a known lineage. Members of the genus *Macavirus* are well recognized as natural infections of ruminants, with several species causing malignant catarrhal fever (MCF) following cross-species transmission to susceptible hosts [46]. The bacterial communities detected in dorcas gazelles were dominated by taxa commonly associated with the gastrointestinal and upper respiratory tracts. Although several of these bacteria are recognized opportunistic pathogens capable of causing septicemia, pneumonia, or enteric disease under predisposing conditions, their concurrent detection in multiple carcasses does not support a single bacterial etiology for the mortality event. Instead, the predominance of enteric-associated microorganisms, together with the detection of environmental and opportunistic bacteria, is more consistent with postmortem bacterial translocation and microbial overgrowth, phenomena that substantially alter microbial communities following death [47].

Multiple viral gene annotations in both Dorcas gazelle and European bison were assigned to parapoxviruses, with the majority of them specific to ORF virus (ORFV). This virus is known as an important pathogenic agent of small ruminants, especially goats and sheep, causing skin lesions including papules and pustules that could develop into ulcerating scabs [48]. The pathological characteristics of parapoxvirus infection are consistent with several features observed in the early stages of NBP of European bison bulls; therefore, the preliminary results seemed very promising. Consequently, the detection of poxvirus DNA in genital lesions expands the spectrum of potential pathogens associated with balanoposthitis and raises the hypothesis that viral infection may either initiate epithelial damage or act synergistically with bacterial pathogens to promote chronic inflammation and tissue necrosis. Although no previous data showed infections with it in either Dorcas gazelle or European bison, it was shown to infect also larger ruminant species such as camels, musk ox, caribou, Sitka black-tailed deer or reindeer [16,49,50]. Two of the European bison tested in our study showed necrotic balonpostitis, a type of lesion that may have been associated with parapoxvirus infections in bovids [51]. Notably, pseudocowpox virus has been associated with ulcerative vulvovaginitis in dairy cattle, and molecularly confirmed infection has also been described in American bison (*Bison bison*), where lesions affected the vulva, udder, inguinal skin, and adjacent perineal tissues [18]. Therefore, these observations demonstrate that parapoxviruses are capable of infecting genital and perigenital epithelia in bovids, supporting the biological plausibility that related viruses may also infect the penis and prepuce of European bison. Parapoxvirus-related annotations represented an instructive example of the limitations of automated metagenomic taxonomic assignment. ORFV-related annotations were detected in both Dorcas gazelle and European bison datasets; however, subsequent analysis did not support authentic ORFV infection. Scaffold alignments were restricted to two localized regions of the ORFV D1701 (HM133903.1) reference genome and involved three annotated proteins (PP1, PP287 and PP288), rather than showing distributed coverage across the viral genome. Moreover, ORFV-specific PCR assays targeting two independent genomic loci were negative in all samples. One of the regions producing metagenomic matches also showed extensive similarity to mammalian genomic sequences. Taken together, these observations are consistent with the possibility that the ORFV-associated annotations resulted from sequence homology involving host-derived DNA and limited regions of the viral reference genome, rather than from the presence of an authentic parapoxvirus genome. Poxvirus genomes are known to contain genes of host origin acquired during evolution through horizontal gene transfer, many of which are involved in modulation of host immune response and contribute to viral pathogenicity [52,53]. Therefore, the presence of regions within the D1701 reference genome showing similarity to mammalian genomic sequences provides a biologically plausible explanation for the observed metagenomic matches. Finally, an alternative explanation that should be considered would be the inadvertent incorporation of host genomic DNA into the original D1701 reference sequence during genome assembly. While the available data do not allow verification of this possibility, the absence of comparable matches in other ORFV reference genomes is consistent with such a scenario. Regardless of the underlying mechanism, those observations illustrate potential pitfalls of reference-based metagenomic analyses, where conserved or host-derived sequences may generate misleading taxonomic assignments if not verified by genome-wide mapping or independent molecular assays such as PCR tests.

Furthermore, the analysis of the bacterial metagenome of the foreskin has also revealed some novel findings. In the two bulls affected by NBP, the predominance of *Porphyromonas levii*, together with *Peptoniphilus* spp. and *Neofamilia massiliensis*, is consistent with a polymicrobial anaerobic microbiota typically associated with chronic necrotic soft-tissue infections; however, it has never been described before in cases of NBP. Nevertheless, *Porphyromonas levii* has been implicated in bovine necrotic and suppurative conditions, including dermatitis, metritis, and genital infections, including in European bison [22,23,54]. Similarly, *Peptoniphilus* species are opportunistic anaerobes frequently recovered from chronic wounds and abscesses in both humans and animals, contributing to endometritis in cattle [55]. The absence of a single dominant pathogen common to all affected animals further supports the hypothesis that bacterial infection is primarily opportunistic and develops following disruption of the genital mucosal barrier. However, because only two affected animals were examined and samples from unaffected preputial tissue were unavailable for comparison, the present data cannot determine whether these bacteria initiated the lesions or preferentially colonized tissue following mucosal disruption. However, the present study provides the first culture-independent, high-throughput sequencing-based characterization of the genital microbiota in NBP-affected European bison, revealing a previously unrecognized dominance of *Porphyromonas levii* and *Peptoniphilus*-associated anaerobes. Consequently, the predominance of anaerobic taxa observed here likely reflects their role in lesion progression and maintenance rather than serving as primary etiological agents, leaving open the possibility that other infectious agents, including viruses, may contribute to disease initiation.

In contrast to the polymicrobial bacterial communities associated with necrotic balanoposthitis, lung samples collected from European bison that died of severe pneumonia were overwhelmingly dominated by *Pasteurella multocida*, supporting its role as the principal bacterial pathogen in these fatal respiratory infections. It is consistent with previous reports identifying this bacterium as an important respiratory pathogen in European bison and a frequent cause of severe bronchopneumonia, particularly in animals with predisposing factors or concurrent infections [56,57]. The predominance of *P. multocida* among bacterial gene annotations observed in the present study is consistent with its potential involvement in the observed fatal pneumonia. Environmental and climatic changes are increasingly recognized as important drivers of pasteurellosis outbreaks in wildlife by promoting physiological stress, impaired immunity, and increased pathogen transmission [58]. Large mortality events associated with *Pasteurella multocida* have been linked to drought and heat stress in several species, including the saiga antelope (*Saiga tatarica*), where climatic conditions triggered hemorrhagic septicemia and mass mortality [59]. Similar mechanisms may increase the susceptibility of European bison to respiratory pasteurellosis, highlighting the importance of integrating disease surveillance with environmental monitoring under changing climatic conditions. Other bacteria were represented predominantly by environment-associated taxa and occurred at substantially lower numbers of gene annotations, making their interpretation as primary respiratory pathogens less supported by the present data. These organisms are commonly associated with soil, freshwater, and plant-associated environments and have only rarely been implicated in disease, suggesting that their presence most likely reflects environmental contamination, postmortem translocation, or incidental components of the pulmonary microbiota rather than etiologically significant pathogens. Collectively, these findings demonstrate a clear distinction between the microbial profiles of respiratory and genital lesions in European bison, whereas fatal pneumonia was associated with the predominance of a recognized bacterial pathogen; necrotic balanoposthitis exhibited a heterogeneous polymicrobial anaerobic community lacking a consistently dominant etiological bacterium. This contrast further supports the hypothesis that bacteria play different roles in the pathogenesis of these conditions and that, in the case of balanoposthitis, additional infectious agents, including viruses, may contribute to disease initiation.

## 5. Conclusions

Metagenomic sequencing performed in the study showed the potential of this method as a tool for investigating disease outbreaks in wildlife by enabling the simultaneous detection of viral and bacterial agents. However, several limitations should be considered when interpreting the results, including the lack of host-read removal for *Gazella dorcas* samples, the relative nature of abundance estimates, and the potential for erroneous taxonomic assignments based on sequence similarity to reference genomes. Additionally, as sequencing control results were not available, contamination cannot be completely excluded. Nevertheless, the approach provided molecular evidence for the presence and further characterization of a previously identified Macavirus in Dorcas gazelles, identified distinct bacterial communities in European bison affected by pneumonia and necrotic balanoposthitis, and generated new hypotheses regarding the multifactorial etiology of these diseases. These findings highlight the potential of mNGS to complement conventional diagnostics and improve diagnostics and pathogen surveillance in wildlife populations.

## Figures and Tables

**Table 1:** Primers used for confirmatory PCR reactions.

Specificity	Target Gene	Primer	Sequence (5′-3′)	Amplicon Size (bp)	Reference
Orf Virus	vIL-10	vIL-10-3	ATGCTACTCACACAGTCGCTC	300	[16]
		vIL-10-4	TATGTCGAACTCGCTCATGGCC		
	GIF	GIF-5	GCTCTAGGAAAGATGGCGTG	408	
		GIF-6	GTACTCCTGGCTGAAGAGCG		

## Data Availability

Partial genomic sequence of Gazella dorcas gammaherpesvirus 2 analyzed in this study is available in NCBI GenBank under accession number PZ867132. The raw sequencing data available for this study have been deposited in the NCBI Sequence Read Archive (SRA) under BioProject accession number PRJNA1503395. Other data that support the findings of this study are available from the corresponding author upon reasonable request.

## Supplementary Materials

The following supporting information can be downloaded at: https://www.mdpi.com/article/10.3390/v18101118/s1, Table S1: Sequencing and quality-control statistics of the analyzed samples; Table S2: Quality of samples chosen for shotgun-based metagenomic analysis; Table S3: Kingdom-level distribution of annotated genes (unigenes) from Dorcas gazelle and European bison samples; Table S4: Most frequent Retroviridae-related annotations identified in Dorcas gazelle metagenomic datasets; Table S5: Annotated viral CDS in the reconstruction of Gazella dorcas gammaherpesvirus 2 isolate PL/2023/Zamosc/dorcas/3B/GDGHV2 partial genome sequence; Table S6: Metagenomic scaffolds contributing to the reconstruction of the Gazella dorcas gammaherpesvirus 2 isolate PL/2023/Zamosc/dorcas/3B/GDGHV2 partial genome sequence.

## Abbreviations

The following abbreviations are used in this manuscript:

NGS	next-generation sequencing
SARS-CoV-2	severe acute respiratory syndrome coronavirus 2
NBP	necrotic balanoposthitis
MEM	Minimum Essential Medium
RPK	reads per kilobase
ORF	open reading frame
ORFV	Orf virus
PCR	polymerase chain reaction
DPOL	DNA polymerase
TAE	Tris-acetate-EDTA
GIF	glycosylation-inhibiting factor
mNGS	metagenomic next-generation sequencing
EVEs	endogenous viral elements
MCF	malignant catarrhal fever
