# Supplementary material for: Next-Generation Sequencing in Practical Wildlife Disease Investigation: Metagenomic Insights into European Bison and Gazelle Mortality and Morbidity Events
Source: Viruses. 2026 Oct 9;18(10):1118. doi: 10.3390/v18101118 (PMC13709774; doi:10.3390/v18101118)
Supplement: Supplementary file 1 [file viruses-18-01118-s001.zip › viruses-4568619-supplementary.pdf]

Table S1 Sequencing and quality-control statistics of the analyzed samples.

Animal No.	Sample code	Raw data (Gb*)	Clean data (Gb)	Clean data (%)	Non-host data (Gb)
1.	A123K	9.32	9.15	98.20	2.79
	A123S	8.94	8.83	98.78	2.59
2.	A126K	8.19	8.06	98.45	2.56
	A126N	9.43	9.29	98.49	2.96
3.	A158K	7.46	7.31	97.90	2.53
4.	A1018	8.88	8.80	99.10	0.08
5.	A1019	8.28	8.21	99.16	0.04
6.	A1162	9.07	9.01	99.33	0.05
7.	A1163	9.64	9.56	99.17	0.03

Note: *Gb, gigabases, corresponds to 10^9 sequencing bases. Raw, clean and non-host data are reported as sequencing bases, as provided by the sequencing provider. Host-read filtering was performed using the host reference genomes available to the sequencing provider. Because a suitable *Gazella dorcas* reference genome was unavailable, reads derived from *G. dorcas* could not be removed. Data were obtained from the sequencing and bioinformatics report provided by Novogene GmbH and compiled by the authors.

Table S2. Quality of samples chosen for shotgun-based metagenomic analysis.

**Animal No.**	**Sample code**	**Host**	**Sample type**	**Total DNA amount (μg)**	**Concentration ng/ul**	**Sample QC results**
1.	A123K	*Gazella dorcas*	blood	0.83273	97.97	Pass
	A123S	*Gazella dorcas*	spleen	2.65491	156.17	Pass
2.	A126K	*Gazella dorcas*	blood	1.27193	57.82	Pass
	A126N	*Gazella dorcas*	nasal swab	0.45257	21.55	Pass
3.	A158K	*Gazella dorcas*	blood	1.65997	103.75	Pass
4.	A1018	*Bison bonasus*	foreskin	23.70081	607.71	Pass
5.	A1019	*Bison bonasus*	foreskin	9.15447	234.73	Pass
6.	A1162	*Bison bonasus*	lung	21.22464	573.64	Pass
7.	A1163	*Bison bonasus*	lung	21.87253	560.83	Pass

Table S3. Kingdom-level distribution of annotated genes (unigenes) from Dorcas gazelle and European bison samples.

Kingdom	A123K		A123S		A158K		A126K		A126N		A1018		A1019		A1162		A1163	
	N	%	N	%	N	%	N	%	N	%	N	%	N	%	N	%	N	%
Archaea	13	0.01	11	0.01	15	0.01	10	0.01	8	0.004	8	0.05	4	0.04	0	0	0	0
Bacteria	85,154	45.06	86,263	45.00	76,395	43.37	80,632	43.98	94,800	48.04	13,788	87.08	9,676	86.84	9,753	89.35	9,353	89.11
Eukaryota	740	0.39	705	0.37	672	0.38	687	0.37	689	0.35	17	0.11	20	0.18	24	0.22	22	0.21
Viruses	4,635	2.45	4,655	2.43	4,668	2.65	4,673	2.55	4,648	2.36	88	0.56	52	0.47	24	0.22	25	0.24
Unassigned	98,421	52.08	100,070	52.20	94,416	53.59	97325	53.09	97,173	49.25	1,933	12.21	1,390	12.48	1,114	10.21	1,096	10.44
**Total**	**188,963**	**100.00**	**191,704**	**100.00**	**176,166**	**100.00**	**183,327**	**100.00**	**197,318**	**100.00**	**15,834**	**100.00**	**11,142**	**100**	**10,915**	**100.00**	**10,496**	**100.00**

Table S4. Most frequent Retroviridae-related annotations identified in Dorcas gazelle metagenomic datasets**.** The table summarizes the closest Reteroviridae annotated GenBank matches obtained during taxonomic assignment. Identified annotations were distributed across multiple retroviral taxa originating from a broad range of vertebrate hosts and included representatives of both Gamma- and Betaretrovirus genera.

Closest annotated match	GenBank accession	Number of matches	Host	Genus
Murine leukemia virus	AAB58752.1	2711	Rodent	Gammaretrovirus
Bat gammaretrovirus	AFA52560.1	2401	Bat	Gammaretrovirus
Reticuloendotheliosis virus	UUG60161.1	2326	Bird	Gammaretrovirus
Porcine type-C oncovirus	QDH76136.1	2307	Pig	Gammaretrovirus
Murine leukemia-related retroviruses	ADU55771.1	1622	Rodent	Gammaretrovirus
Koala retrovirus	BAM67146.1	668	Koala	Gammaretrovirus
Feline leukemia virus	QXN66309.1	618	Cat	Gammaretrovirus
Baboon endogenous virus	YP_009109695.1	543	Baboon	Gammaretrovirus
RD114 retrovirus	BAM17307.1	535	Cat	Gammaretrovirus
Gibbon ape leukemia virus	CAA31582.1	503	Gibbon	Gammaretrovirus
Human endogenous retrovirus K	CAA76882.1	457	Human	Betaretrovirus
Mouse mammary tumor virus	AAC24859.1	428	Rodent	Betaretrovirus
Recombinant M-MuLV_RaLV retrovirus	AAO46145.1	425	Rodent	Gammaretrovirus
Jaagsiekte sheep retrovirus	ABI50238.1	423	Sheep	Betaretrovirus
Ovine enzootic nasal tumor virus	AAL76420.1	282	Sheep	Betaretrovirus
Bovine retrovirus CH15	UKS89487.1	266	Cattle	Betaretrovirus
Mason-Pfizer monkey virus	AAD43261.1	246	Primate	Betaretrovirus
Enzootic nasal tumor virus 2	QDF21376.1	218	Sheep	Betaretrovirus
Hervey pteropid gammaretrovirus	QJT93246.1	150	Bat	Gammaretrovirus
Gammaretrovirus sp.	WHP37832.1	145	Undefined	Gammaretrovirus

Table S5. Annotated viral CDS in the reconstruction of *Gazella dorcas* gammaherpesvirus 2 isolate PL/2023/Zamosc/dorcas/3B/GDGHV2 partial genome sequence.

CDS	CDS annotation	Genomic range[a]	Orientation	Status	Mean read depth (x)[c]
1	Cytoplasmic envelopment protein 1	18117–18857	Minus	Partial[b]	472.44
2	Portal protein	18868–20601	Plus	Complete	492.20
3	DNA replication helicase	20600–22924	Plus	Complete	494.37
4	Shutoff alkaline exonuclease	26666–28123	Plus	Complete	510.21
5	Envelope glycoprotein H	30091–31989	Plus	Partial	535.51
6	ORF23	31981–33207	Minus	Complete	541.78
7	Major capsid protein	35419–39522	Plus	Complete	527.41
8	Triplex capsid protein 2	39548–40462	Plus	Complete	491.25
9	Tripartite terminase subunit 3	41437–42495	Minus	Complete	534.87
10	ORF30	42597–42857	Plus	Complete	513.44
11	ORF31	42854–43453	Plus	Complete	469.40
12	Cytoplasmic envelopment protein 2	44822–45853	Plus	Complete	472.41
13	ORF34	46753–47781	Plus	Complete	522.52
14	DNA polymerase	70537–73566	Minus	Complete	550.59
15	Glycoprotein B	73745–76120	Minus	Partial	526.97
16	Major DNA-binding protein	78514–81918	Minus	Complete	544.83

Note: [a]Genomic range refers to the reconstructed sequence. [b]"Partial" indicates incomplete CDS boundaries due to insufficient homologous sequence support.[c]Mean read depth was calculated from per-base coverage obtained by mapping raw A126N reads to the PX942233.1 reference sequence. All 16 CDS showed 100% nucleotide coverage across their annotated ranges.

Table S6. Metagenomic scaffolds contributing to the reconstruction of the Gazella dorcas gammaherpesvirus 2 isolate PL/2023/Zamosc/dorcas/3B/GDGHV2 partial genome sequence.

Scaffold	Scaffold range[a]	Reference range[b]	Used range[c]	Orientation	Identity (%)	Alignment length (bp)[d]	Inserted length (bp)[e]
A126N_k127_408468_1_87897	9777–28903	28872–47991	28872–47991	Minus	62.669	19504	19120
A126N_k127_283434_1_31255	1492–18470	66935–83696	66935–83696	Plus	61.488	17335	16762
A126N_k127_408468_1_87897	35268–40336	18074–23117	18074–23117	Minus	65.791	5139	5044
A126N_k127_408468_1_87897	4935–8913	48750–52624	48750–52624	Minus	61.078	4026	3875
A126N_k127_408468_1_87897	30020–31975	26346–28296	26346–28296	Minus	66.280	1984	1951
A126N_k127_408468_1_87897	32206–33365	25078–26233	25078–26233	Minus	67.179	1170	1156
A126N_k127_408468_1_87897	41140–42247	16187–17291	16187–17291	Minus	64.711	1125	1105
A126N_k127_408468_1_87897	57343–58430	112–1199	112–1199	Minus	61.006	1113	1088
A126N_k127_255860_1_1133	120–424	96472–96776	96472–96776	Plus	62.997	327	305
A126N_k127_506134_1_1496	670–993	96480–96827	96777–96803	Minus	60.227	352	27
A126N_k127_567656_1_644	181–563	96501–96876	96804–96876	Plus	60.804	398	73
A126N_k127_481291_1_758	382–701	97240–97540	97240–97540	Minus	60.606	330	301
A126N_k127_517212_1_553	11–329	96440–96780	96440–96471	Plus	60.511	352	32

Note: Only scaffolds contributing to the final reconstruction are shown. PX942233.1 partial genome sequence was used as the reference. [a]scaffold coordinates; [b]corresponding PX942233.1 coordinates; [c]reference coordinates incorporated into the reconstruction; [d]BLASTN alignment length; [e]number of nucleotides from the scaffold contributing to the final reconstruction.
